# Longitudinal effects of bushfire harm on adolescent mental health

**DOI:** 10.1177/00048674251413876

**Published:** 2026-01-19

**Authors:** Anton T. du Toit, Kate Maston, Andrew Mackinnon, Alison Calear, Bridianne O’Dea, Michelle Torok, Aliza Werner-Seidler

**Affiliations:** 1 Translational Health Research Institute, Western Sydney University, Penrith, NSW, Australia; 2 Black Dog Institute, University of New South Wales Sydney, Sydney, NSW, Australia; 3 Flinders University, Adelaide, SA, Australia; 4 School of Psychology, University of New South Wales Sydney, Sydney, NSW, Australia

**Keywords:** Bushfires, wildfires, mental illness, adolescents, longitudinal study, cohort study

## Abstract

**Background:**

In Australia, harms associated with bushfires are expected to increase as the severity and frequency of bushfires increase with climate change. Bushfire harm includes negative impacts on mental health, particularly for adolescents. Evidence suggests that elevated incidence of mental illness can persist long-term after bushfire harm. This study extends our team’s earlier cross-sectional analysis within the same cohort. It examines effects of the Black Summer bushfires (2019-2020) on adolescents’ mental health measured prospectively and explores risk and protective factors associated with sustained mental health problems following bushfire harm.

**Methods:**

A broadly representative sample of 2967 Australian adolescents was recruited in 2019-2022 at age 13-14 and followed for 24 months. Bushfire exposure and harm, and symptoms of depression, anxiety, psychological distress, insomnia, and suicidality were measured. Linear regression models examined the effects of bushfires over time; logistic regression identified predictors of these effects.

**Results:**

167 (5.4%) participants reported bushfire harm. Bushfire harm was not a significant predictor of any mental health outcomes at 24 months. Baseline symptoms were most strongly associated with 24-month outcomes. Participants who were gender or sexuality diverse, reported adverse childhood experiences, or had a history of mental health problems had increased risk of symptoms of depression, anxiety, distress and insomnia at 24-month follow-up compared to those who did not have these risk factors.

**Conclusions:**

Bushfire-harmed adolescents did not show significantly elevated mental health symptoms compared to unexposed peers 24 months later. This finding is encouraging, though its underlying causes are unclear and require further research.

## Introduction

The frequency and severity of natural disasters has been increasing over the past 50 years. The International Disaster Database recorded 81 natural disasters globally in 1970, rising to 399 in 2023 (CRED, 2024). Based on a linear projection of these trends, by 2030 there could be 560 disasters per year (United Nations Office for Disaster Risk Reduction, 2022). In Australia, the number of people affected by natural disasters has been trending upwards since 2000 (Delforge et al., 2025).

Natural disasters have been shown to typically increase the prevalence of mental illness in adult and adolescent populations; not only post-traumatic stress, but also other anxiety disorders and depression (Beaglehole et al., 2018; Newnham et al., 2022; Tang et al., 2014; Weilnhammer et al., 2021). The effects of natural disasters on mental health depend on the nature of the disaster itself, the population, and level of personal exposure to the disaster. For example, increased severity of the disaster and repeated exposures to natural disasters have been shown to be associated with worse mental health outcomes (Goenjian et al., 2021; Harville et al., 2018; Mitchell et al., 2024; Sugg et al., 2023).

Adolescents seem to be more affected by disasters than adults. A comprehensive review which included 234 studies showed that after disasters, adolescents experience higher rates of depression and anxiety relative to adults, both immediately after the disaster and in the years following (Newnham et al., 2022). Disaster harm may disproportionally impact adolescents relative to adults via multiple mechanisms. First, this might be due to impacts on the young person’s physical health at a developmentally critical time for growth and development, via disruptions to food access, clean water and medical care (Kousky, 2016). Further, there may be adverse effects on young people via disruption to their home environment if they are displaced, or via impact on their parent or carer’s stress, mental health, and parenting (Cobham et al., 2016). The literature suggests that the need to relocate can be particularly difficult for children and adolescents following disaster (Hansel et al., 2013). Moreover, disasters often interrupt education and schooling, which can have long term negative effects on academic outcomes (Gibbs et al., 2019). Collectively, this evidence suggests that outcomes for children and adolescents following disaster exposure might be particularly pronounced; an assertion supported by the literature (Newnham et al., 2022).

Bushfires are a critically important area of investigation in the Australian context. Australia has 0.33% of the world’s population, yet since 2000 has had 9.79% of deaths due to bushfires (Central Intelligence Agency, 2024; CRED / UCLouvain, 2024). Further, Australia has a recent history of major bushfires, a steady trend upwards in the number of people affected by them, and the likelihood of more severe bushfires in the future (CSIRO, 2024). Yet there are relatively few studies relating specifically to the mental health effects of bushfires, and even fewer on their relationship to young peoples’ mental health. In a comprehensive review of the long-term mental health effects of disasters and pandemics conducted in 2022 (Newnham et al., 2022), there were 82 studies examining child and adolescent populations and 17 examining mixed-age populations – yet none of these studies assessed the effects of bushfires. A 2023 review of the effects of bushfires and wildfires upon health included five studies focused on mental health in children or adolescents, but these studies covered only two events and were drawn from only two data sets (Gao et al., 2023). Both had large samples; one event was the Canadian Fort McMurray fires in 2016 (baseline *n* = 3070, out of 4407 students in the fire-affected adolescent population), and the other event was the Ash Wednesday bushfires in South-Eastern Australia (baseline *n* = 808, drawn from six schools attended by children affected by the fires).

Several studies of bushfires and mental health show that among affected adolescent populations the incidences of mental illnesses (other than trauma-related disorders) may remain at an elevated level or even increase. A controlled study of children exposed to the 1983 Ash Wednesday bushfires in South Australia found significantly higher point prevalences of any DSM-IV diagnosis (15.2% compared to 11.0%, RR 1.42, 95% CI (1.02–1.98), *p* = 0.04) and of anxiety disorders excluding PTSD (10.8% compared to 6.9%, RR 1.66, 95% CI (1.09–2.53), *p* = 0.02) in the bushfire-affected group after 20 years (McFarlane and Van Hooff, 2009). A cross-sectional study with a non-affected control group showed elevated rates of symptoms in high school students affected by the 2016 Fort McMurray wildfire in Alberta, Canada, 18 months after it occurred (Brown et al., 2019). Further, repeated cross-sectional samples of high school students affected by that fire showed that symptoms of mental health problems increased from 18 months after the wildfire to 42 months later. Assessed using clinical cut-off scores, the rate of probable depression increased from 31% in 2017 to 35% in 2019 (*p* = .00033), and the rate of probable anxiety increased from 27% in 2017 to 31% in 2019 (*p* = .00020) (Brown et al., 2021).

Although these two studies are informative, compared to prospective cohort designs they are limited by the use of matched controls, in that unexplained between-subject variation is present. In contrast, prospective cohort designs eliminate this unexplained variation by sampling the same participants at two time points (Batterham et al., 2024; Mitchell et al., 2024). The present study aims to investigate the effects of bushfires on adolescent mental health using a prospective cohort design involving bushfire-harmed and non-harmed adolescents.

The Black Summer bushfires began in south-eastern Australia in September 2019 and ended in March 2020, having ultimately burned 24.3 million hectares, an area the size of Uganda or the United Kingdom. Thirty-four people died as a direct result of the bushfires with an estimated 417 more dying as a result of smoke inhalation (Borchers Arriagada et al., 2020). We previously found that exposure to harm associated with the Black Summer bushfires was associated with trauma, suicidal ideation, and insomnia in a cross-sectional study of this cohort (Beames et al., 2023).

The aim of this study is to examine the influence of bushfires upon the subsequent mental health of adolescents following the Black Summer bushfires, using data from 24-month follow-up. The first research question is to identify the effect of bushfire harm on mental health in bushfire-harmed adolescents in the years following the Black Summer bushfires. The second research question is to identify demographic, health, and contextual factors that increase or decrease risk of deterioration in mental health in this population following a bushfire.

## Methods

### Sample

The data from this study was drawn from a longitudinal cohort study (The Future Proofing Study). Participants were Australian school students. All participants were in Year 8 at the time of recruitment (96.2% were aged 13 or 14 years; mean 13.84, SD 0.49). Participants were recruited through secondary schools in three cohorts, from March 2019 to March 2022. In total 134 high schools participated, with representation across all school sectors (i.e. government, independent, and Catholic). By design, most schools were located in New South Wales (at baseline, 121 of 134 schools (90.3%); 5373 of 6288 participants; 85.4%), as data collection involved in-person visits by the Sydney-based research team (see cohort profile for more information (Werner-Seidler et al., 2023)).

Data from participants who had completed baseline, 12- and 24-month follow-up assessments for the variables analysed were included. The sample pool was sufficiently large that 1190 cases with missing data in the predictor or outcome variables were removed, resulting in a final sample of *n* = 2967, being 46.4% of the number enrolling in the study at baseline. Of these, 167 had suffered bushfire harm as defined in this paper. Attrition among participants across the period of the study relevant to this paper, and the number excluded due to incomplete data, are detailed more fully in Supplementary Figure 1. Sample demographics at baseline are shown in Table 1, with a comparison of the initial and final samples in Supplementary Table 1.

**Table 1.** Sample characteristics at baseline.

Variable	Harmed by bushfires (*n* = 167)	Not harmed by bushfires (*n* = 2800)	Total at baseline (*n* = 2967)
***Gender***			
Male	45.5% (76)	44.0% (1232)	44.1% (1308)
Female	51.5% (86)	52.0% (1456)	52.0% (1542)
A different gender	2.4% (4)	2.9% (81)	2.9% (85)
Prefer not to say	0.6% (1)	1.1% (31)	1.1% (32)
***Age at baseline***			
11 years	0.0% (0)	0.1% (2)	0.1% (2)
12 years	3.6% (6)	3.9% (109)	3.9% (115)
13 years	51.5% (86)	53.8% (1506)	53.7% (1592)
14 years	44.9% (75)	42.2% (1181)	42.3% (1256)
15 years	0.0% (0)	0.1% (2)	0.1% (2)
***State***			
NSW	92.8% (155)	82.9% (2321)	83.5% (2476)
QLD	0.6% (1)	5.0% (140)	4.8% (141)
SA	0.6% (1)	2.0% (55)	1.9% (56)
VIC	3.0% (5)	6.3% (177)	6.1% (182)
WA	3.0% (5)	3.8% (107)	3.8% (112)
***Sexuality***			
Heterosexual or straight	77.8% (130)	74.2% (2078)	74.4% (2208)
Sexuality diverse	12.6% (21)	12.7% (355)	12.7% (376)
Not sure	7.8% (13)	8.6% (240)	8.5% (253)
Prefer not to say	1.8% (3)	4.5% (127)	4.4% (130)
***Symptoms of mental illness***			
Depression symptoms above threshold	13.2% (22)	11.0% (309)	11.2% (331)
Anxiety symptoms above threshold	18.6% (31)	15.5% (434)	15.7% (465)
Distress symptoms above threshold	25.1% (42)	27.4% (766)	27.2% (808)
Insomnia symptoms above threshold	13.8% (23)	8.2% (229)	8.5% (252)
Suicidality symptoms above threshold	7.8% (13)	3.8% (106)	4.0% (119)

Notes: ‘Another gender' is an aggregation of ‘Non-binary’ and ‘Other’.

### Measures

Data was obtained from self-report questionnaires. The instruments used to measure outcome and risk variables are described below.

**Bushfire harm**: Bushfire harm was defined as the participant self-reporting one or more of the following: being evacuated from their home, suffering property damage, or suffering injury, using a measure adapted from a previous study adapted for adolescent populations (Parslow et al., 2006). We focused on these acute forms of bushfire harm given they are likely to reflect a higher level of severity relative to other more distal harms associated with bushfire exposure (e.g., smoke exposure), and evidence indicates that greater harm severity may be associated with worse psychological outcomes (Macleod et al., 2024). The three forms of bushfire harm measured in this study were collapsed into one category because the number of participants who suffered bushfire harm was relatively low and to remain consistent with the previous research on natural disasters we conducted with this cohort (Beames et al., 2023). Descriptive statistics regarding the specific type and number of bushfire harms experienced are provided in Supplementary Table 2. This was in line with our previous bushfire harm research (Beames et al., 2023) and findings from previous studies that mental health effects of disasters generally increase with the severity of individual harm (Goenjian et al., 2021; Sugg et al., 2023).

**Outcomes**: Depression was measured with the Patient Health Questionnaire: Adolescent Version (PHQ-A; clinical threshold ⩾ 15) (Johnson et al., 2002), anxiety with the Spence Children’s Anxiety Scale (SCAS; clinical threshold ⩾ 14) (Spence, 1998), distress with the Distress Questionnaire 5 (DQ5; clinical threshold ⩾ 14) (Batterham et al., 2016), insomnia with the Insomnia Severity Index (ISI; clinical threshold ⩾ 15) (Bastien et al., 2001), suicidal ideation with the Suicidal Ideation Attributes Scale (SIDAS; clinical threshold ⩾ 21 for high risk of suicide behaviour and ideation) (Van Spijker et al., 2014), and bushfire-related trauma with an adapted version of the Child Traumatic Stress Questionnaire (Kenardy et al., 2006). The latter was only administered to participants whose home or school was in an area threatened by the bushfires (Beames et al., 2023).

Standardised Cronbach's alphas at baseline and at 24-month follow-up for the five outcome measures were: PHQ-A .87 and .88, SCAS .93 and .93, DQ5 .87 and .88, ISI .85 and .86, SIDAS .65 and .64.

**Risk factors**: The risk factors included were identical to those in the previous paper (Beames et al., 2023): adverse childhood experiences (measured with the BRFFS-ACE (Centers for Disease Control and Prevention, 2009)), COVID diagnosis or quarantine, and mental illness history, all dichotomised as Yes or No; gender identity, categorised as Male, Female, Another gender, and Prefer not to say; sexual orientation, categorised as Heterosexual, Sexuality diverse, Unsure, and Prefer not to say; perceived household wealth, categorised as High, Low, and Prefer not to say; and language spoken most at home, dichotomised as English and Other.

### Procedure

Participants completed self-report questionnaires on a secure website during class time. Participation required an active Android or iOS-based smartphone (used to collect cognitive task, typing, speech, ecological momentary assessments, and sensor data).

Bushfire exposure and harm questions were included in the baseline data collection.^1^ The mean time elapsed between the end of the bushfire period and data collection was 410 days (SD 141 days). Follow-up assessments were performed at approximately 12-month intervals after the baseline assessment, repeating the outcome measures used at baseline.

Informed consent was obtained from parents/carers and adolescents. The ethical aspects of this research were approved by the NSW Department of Education (SERAP2019201), the Dioceses of the participating Catholic schools, and the UNSW Human Research Ethics Committee (HC180836).

### Data preparation and analysis

A small number of participants mis-stated their date of birth (*n* = 128, or 2.0% of the starting sample), causing their age at entry to be incorrect (i.e., less than 11 or greater than 15 years of age); these values were replaced with the mean age of the cohort (13.9 years) for those in the acceptable range.

The outcomes for the models were dichotomous variables based on the clinical thresholds for the five mental health measures, covering depression, anxiety, distress, insomnia and suicidality. Because of the disparity in times at which the baseline and subsequent questionnaires were completed across the sample (Supplementary Figure 2), the first research question addressed the effect of time elapsed between the bushfires and baseline data collection on the prospective mental health of participants harmed by the bushfires. This analysis used linear regression models relating time elapsed to mental health outcomes. For the second research question, logistic multivariate models were used to examine the relationship between bushfire harm and subsequent mental health problems, and the risk and protective factors involved in this relationship. The models used predictors that are known risk factors for mental illness: the same predictors as those in the previous bushfire harm paper (Beames et al., 2023), with the addition of the baseline measurements of the outcomes.

The power of models to discriminate cases and non-cases was evaluated using the Area Under Curve (AUC), with an AUC of 0.5 indicating chance level performance and a value of 1.0 perfect discrimination. Data processing, visualisation and analysis was performed in R and associated libraries (Baillie et al., 2024; R Core Team, 2024).

## Results

### Bushfire harm

Among the 167 participants reporting bushfire harm, 123 (73.7%) were evacuated from their homes; 16 (9.6%) had their homes or possessions damaged or destroyed, and 54 (32.3%) were injured (refer to Supplementary Table 2 for more detail).

### Effect of time elapsed since the fires on mental health in the bushfire-harmed population (RQ1)

The period between the bushfires and baseline questionnaire completion varied considerably, ranging from 179 to 681 days after the most intense phase of the bushfires in December 2019 to January 2020 (mean 410 days, SD 141; Supplementary Figure 2). The first research question related only to the bushfire-harmed subsample; it was whether the time elapsed between the fires and baseline assessment predicted mental health outcomes. We used linear regression models relating the five mental health outcomes and bushfire trauma to the number of days from the bushfires to baseline data collection. Time elapsed was not a significant predictor for any of these outcomes (Depression: β = −0.03, 95% CI [−0.12, 0.06]; Anxiety: β = −0.05, 95% CI [−0.14, 0.04]; Distress: β = −0.03, 95% CI [−0.12, 0.06]; Insomnia: β = −0.07, 95% CI [−0.16, 0.02]; Suicidality: β = −0.02, 95% CI [−0.12, 0.07]; Bushfire trauma: β = −0.07, 95% CI [−0.16, 0.02]; β is standardised beta).

### Effects of bushfires on mental health (RQ2)

Bushfire harm did not reach significance as a predictor for any of the five mental health outcomes at 24-month follow-up, as shown in Table 2.

**Table 2.** Forced-selection models of 24-month mental health outcomes (odds ratios).

	Depression symptoms	Anxiety symptoms	Distress symptoms	Insomnia symptoms	Suicidality symptoms
Parameter	Coefficient (95% CI)	Coefficient (95% CI)	Coefficient (95% CI)	Coefficient (95% CI)	Coefficient (95% CI)
***Baseline outcome***					
Above threshold at baseline	4.34*** (3.22, 5.83)	5.33*** (4.12, 6.89)	4.28*** (3.51, 5.21)	4.92*** (3.58, 6.74)	6.20*** (3.48, 10.84)
***Gender identity***					
Gender identity – another gender or prefer not to say	2.55*** (1.49, 4.34)	3.72*** (2.14, 6.42)	3.44*** (2.17, 5.50)	2.29** (1.21, 4.19)	1.97 (0.86, 4.40)
Gender identity – female	1.94*** (1.47, 2.59)	3.12*** (2.32, 4.23)	4.07*** (3.35, 4.97)	2.59*** (1.95, 3.47)	1.26 (0.74, 2.20)
***Sexuality***					
Sexuality – diverse	1.53** (1.11, 2.09)	1.13 (0.82, 1.54)	1.29* (1.00, 1.64)	0.80 (0.56, 1.12)	3.00*** (1.70, 5.21)
Sexuality – not sure	1.32 (0.86, 1.97)	1.08 (0.72, 1.59)	1.49** (1.10, 2.01)	0.92 (0.59, 1.41)	2.56** (1.18, 5.16)
***Other demographics***					
Do not speak English at home	0.81 (0.47, 1.34)	0.58* (0.32, 0.97)	0.67* (0.45, 0.96)	1.00 (0.59, 1.61)	0.63 (0.18, 1.68)
Perceived SES – low	1.32* (1.01, 1.72)	1.23 (0.96, 1.59)	1.14 (0.94, 1.38)	1.58*** (1.21, 2.07)	1.12 (0.68, 1.86)
Perceived SES – prefer not to say	1.05 (0.69, 1.57)	1.11 (0.75, 1.62)	1.21 (0.91, 1.59)	1.44 (0.96, 2.12)	1.05 (0.48, 2.16)
***Mental health and trauma***					
MH diagnosis – any	1.59*** (1.17, 2.14)	1.62*** (1.20, 2.16)	1.47*** (1.15, 1.89)	1.47* (1.07, 2.01)	2.64*** (1.60, 4.30)
ACEs – at least one	1.59*** (1.17, 2.18)	1.42** (1.08, 1.90)	1.22* (1.00, 1.49)	1.57*** (1.17, 2.13)	2.10* (1.09, 4.47)
COVID – diagnosed or quarantined	1.15 (0.81, 1.62)	0.98 (0.69, 1.37)	1.02 (0.79, 1.33)	1.03 (0.71, 1.46)	1.10 (0.57, 2.00)
Bushfire harm – yes	0.73 (0.40, 1.26)	1.08 (0.65, 1.74)	1.25 (0.86, 1.81)	0.60 (0.32, 1.05)	0.88 (0.29, 2.14)
***Model predictive power***					
AUC	0.75	0.81	0.79	0.73	0.81

Notes: Predictors have been included in the table if they are significant for any of the five outcomes.

* Significant at *p* < 0.05. ** Significant at *p* < 0.01. *** Significant at *p* < 0.001.

‘Baseline outcome – Above threshold at baseline’ means that the participant was over the clinical threshold for the condition in that column at the time of their baseline data collection.

In Gender identity, the categories ‘another gender’ and ‘prefer not to say’ were collapsed because of small cell sizes; both had elevated rates of mental illness compared to male and female.

AUC: area under curve. ACEs: adverse childhood events. SES: socioeconomic status.

Reference categories: Baseline outcome – below threshold, Gender identity – male, Sexuality – heterosexual, Do speak English at home, Perceived SES – high, MH diagnosis – none, ACEs – none, COVID – neither diagnosed nor quarantined, Bushfire harm – no.

Table 2 shows odds ratios, i.e. the odds of a person in the category listed being above the clinical threshold for the outcome, relative to a person who is in the reference category; for example, a female was 1.94 times more likely to be over the clinical threshold for Depression relative to a male, with the 95% confidence interval from 1.47 times to 2.59 times more likely. The AUC for the model of Depression was 0.75, indicating that the model had a 75% chance of correctly classifying a person as above the clinical threshold for Depression, given knowledge of the predictor variables. Standard errors for the coefficients are provided in Supplementary Table 3.

The strongest predictors of the five mental health outcome variables measured at 24-month follow-up were the baseline measurements of that variable. These were stronger than other predictors except for Distress, where the coefficients for baseline distress (OR 4.276, 95% CI [3.512, 5.214]) and being female (OR 4.071, 95% CI [3.348, 4.968]) were not significantly different.

The broad patterns of other predictors across Depression, Anxiety, Distress, and Insomnia were similar: gender identity (both female and another gender) was a significant predictor, with previous mental health diagnosis, adverse childhood experiences, both diverse and ‘not sure’ sexuality (for Depression and Distress), and perceived SES (for Insomnia) also having predictive value. All of these variables predicted higher risk; only ‘Do not speak English at home’ predicted lower risk, for Anxiety and Distress.

The pattern for Suicidality was markedly different from the other four outcomes. While the baseline measurement of the outcome was still the strongest predictor, sexuality was the second-strongest, followed by previous mental health diagnosis and adverse childhood experiences. Gender identity was not significant.

Interestingly, a possible recovery effect is evident. Specifically, for each of the three mental health outcomes where bushfire-harmed participants were *more* likely to be above the clinical threshold at baseline (i.e., depression, insomnia and suicidality), they were *less* likely to be above the clinical threshold at 24-month follow-up when adjusted for baseline levels of the outcome (Supplementary Table 4). While this effect did not reach statistical significance, the differences in odds ratios is potentially clinically significant.

## Discussion

The first research question of this study was to examine the effects of bushfire harm on adolescent mental health over time. No significant effect for time elapsed since the bushfires was found for depression, anxiety, distress, insomnia, or suicidal ideation. This suggests that over the period examined in this analysis, i.e. from six months to two years after the bushfires ended, the period of time elapsed did not affect mental health outcomes for the subgroup affected by the bushfires.

The second research question was to identify demographic, health, and contextual factors that increase or decrease risk of deterioration in mental health in this population following bushfire harm. Using logistic regression models, we showed that the bushfires were not a significant predictor of mental health outcomes over the period of the study; rather, the strongest predictors across most of the prospective mental health outcomes were baseline measurements of the outcome, gender, sexuality, adverse childhood experiences, and previous mental health diagnosis – in other words, the same factors associated with increased or decreased risk in the general population.

Bushfire harm did not have ongoing negative mental health effects over the two years following baseline data collection. This was somewhat unexpected, particularly given that the Black Summer fires were severe, the vast majority (>85%) of the sample who reported harm were in the state of NSW which was the most severely fire-impacted state (Filkov et al., 2020), and severity is often associated with worse mental health outcomes (e.g., Macleod et al., 2024). Further, at baseline, an average of 410 days after the fires, there were still mental health differences between bushfire-harmed and unharmed participants in terms of insomnia and suicidality (Supplementary Table 4), so given the results of the Ash Wednesday studies and particularly the Fort McMurray studies, one might have expected these differences to persist (Brown et al., 2021; McFarlane and Van Hooff, 2009). Importantly, our sample was broadly representative of the general population in terms of demographic factors and mental health symptoms prior to the fires. Demographic factors were compared to national census and government data and found to be broadly comparable (Werner-Seidler et al., 2025) and baseline levels of distress, depression and anxiety levels were also in line with other large national surveys of young people (e.g., Australian Institute of Health and Welfare, 2024; Tiller et al., 2021). Notably, there were no differences on these variables at baseline between the bushfire harmed and unharmed samples. Accordingly, the sample included in our study were reflective of the broader adolescent population, enhancing the generalisability of the findings.

These results differ from those reported after the 2016 Fort McMurray fires in Canada, in which mental health symptoms were elevated even three and a half years later (Brown et al., 2021). However, the relative fire severity must be considered: the Fort McMurray fire involved the town’s entire population of 88,000 people being evacuated, 10% of homes destroyed, and 590,000 hectares burned. While the affected areas were larger in the Black Summer fires (i.e., 24.3 million hectares burned), the fires were distributed over a wider area rather than being concentrated in and around one town. The results of this study also differ from those of studies after the Ash Wednesday fires in south-eastern Australia which found worsened mental health two years later (McFarlane et al., 1987). However, the age group was significantly younger (5–12 years), and the measures used were parent- and teacher-rated in this study. There are well documented discrepancies between parent and self-report symptom levels, which may in part account for this difference in findings (De Los Reyes and Kazdin, 2005; Ford and McCoy, 2022). Further, the baseline measurements were taken two months after the fires, whereas in the current study the baseline was on average 13.5 months after. For both of the Ash Wednesday and Fort McMurray fires, the level of response from mental health services is not known, which may have affected the duration and severity of mental health impacts, whereas after the Black Summer bushfires additional mental health resources were made available (NSW Department of Education, 2021; Thomas et al., 2024).

It is not clear why the bushfire-harmed sample in this study did not show adverse mental health effects over the long term. Although speculative, it may have been due to the effects of the bushfire harm dissipating over time without intervention, due to the adolescents’ own resilience and coping skills. This is consistent with previous research which has shown that among young people, following natural disasters, the most common mental health trajectories are that of resilience and recovery (Liang et al., 2021; Shi et al., 2016). These studies (and others) have converged on the findings that variability in symptom trajectories is most common in the 12-18 months following the disaster, and after this time, trajectories tend to stabilise (Fan et al., 2015), which aligns with the period of assessment included in this study. Moreover, factors associated with more resilient trajectories included better social support and higher levels of trait resilience, both of which may offer valuable prevention and disaster readiness targets (e.g., Pinto et al., 2021). Another explanation is that the emergency response to the Black Summer bushfires from the government meant that this subgroup received significant levels of general and mental health support relative to the population (NSW Department of Education, 2021; Thomas et al., 2024). There are a range of psychosocial programs specifically designed for delivery to young people following disaster exposure and harm, with evidence suggesting overall positive effects on depression, anxiety and post-traumatic stress (Brown et al., 2017; Gibbs et al., 2021).

It is also plausible that the communities’ own responses to the bushfires, such as aid and support, provided a coping resource over and above anything delivered by government. This effect in the aftermath of natural disasters has been noted in other studies (e.g., Cretney, 2016).

The predictors of mental health outcomes in the models presented in this paper were ranked similarly to those in the cross-sectional analysis in the earlier paper (Beames et al., 2023). The strongest predictors across the models were demographic variables and not modifiable. They therefore could serve as markers for groups to target for intervention. The markers for the groups of adolescents most vulnerable to mental ill-health in this sample, including the group reporting bushfire harm, were similar to the markers for those who are most vulnerable to mental illness overall (e.g., previous history of mental illness, female gender, gender diversity, adverse childhood experiences, low SES, and/or high neuroticism). Further, because it is largely the same groups of adolescents who are vulnerable to four of the five outcomes studied here, population-level preventive interventions for these groups should seek to cover multiple conditions and be transdiagnostic in nature (Dalgleish et al., 2020). The profile for adolescents at higher risk of suicidality is different, and this group should be considered separately.

There are several strengths of this study that warrant mention, including a large sample of *n* = 2967, broadly representative of the Australian adolescent population, (Werner-Seidler et al., 2023, Table 3) with *n* = 167 exposed and harmed by the bushfires. It is, to the best of our knowledge, the only available longitudinal cohort study of bushfire-affected adolescents which uses a broadly representative sample.

The most significant limitation to this study is that the baseline data collection occurred on average 410 days after the most intense phase of the bushfires had ended (range: 179-681 days; Supplementary Figure 2). Ideally, future research into the effects of bushfires would be conducted as soon as possible after their cessation – but the time required for securing funding and ethics approvals may present a barrier to timely research in this area. It may have been that the majority of the mental health sequelae of the bushfires had passed prior to the initial data collection. Nevertheless, the results still suggest that, at least for the majority of this sample, the mental health ill-effects of the bushfires were relatively short-lived. Whether this was due to the increased resources made available after the bushfires or to other factors may become apparent when there is an evaluation of resource deployment following the Black Summer bushfires. This may help confirm whether support immediately after bushfires is helpful or whether there is a natural process of recovery over time, so that the utility of making more treatment resources available after bushfires may be examined.

## Supplemental Material

sj-docx-1-anp-10.1177_00048674251413876 – Supplemental material for Longitudinal effects of bushfire harm on adolescent mental health Supplemental material, sj-docx-1-anp-10.1177_00048674251413876 for Longitudinal effects of bushfire harm on adolescent mental health by Anton T. du Toit, Kate Maston, Andrew Mackinnon, Alison Calear, Bridianne O’Dea, Michelle Torok and Aliza Werner-Seidler in Australian & New Zealand Journal of Psychiatry

## Notes

1. Except for 216 participants whose baseline data was collected before the Black Summer bushfires; these participants were excluded from the final sample of *n* = 2967.
