# Supplementary material for: Longitudinal effects of bushfire harm on adolescent mental health
Source: Aust N Z J Psychiatry. 2026 Jan 19;60(3):259–68. doi: 10.1177/00048674251413876 (PMC12932682; doi:10.1177/00048674251413876)
Supplement: sj-docx-1-anp-10.1177_00048674251413876 – Supplemental material for Longitudinal effects of bushfire harm on adolescent mental health [file sj-docx-1-anp-10.1177_00048674251413876.docx]

1. Supplementary materials


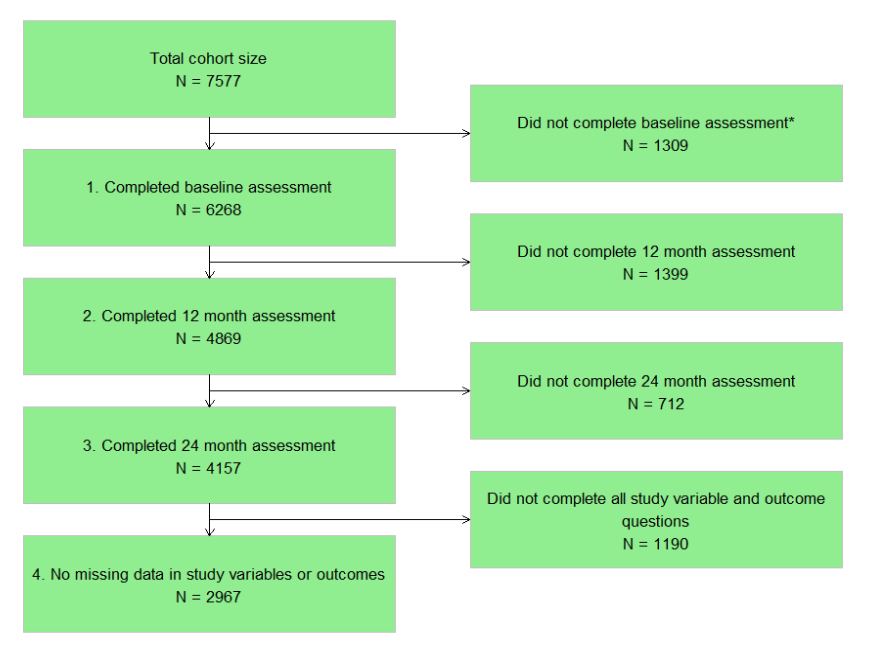


*Supplementary Figure 1: Participant flow diagram*


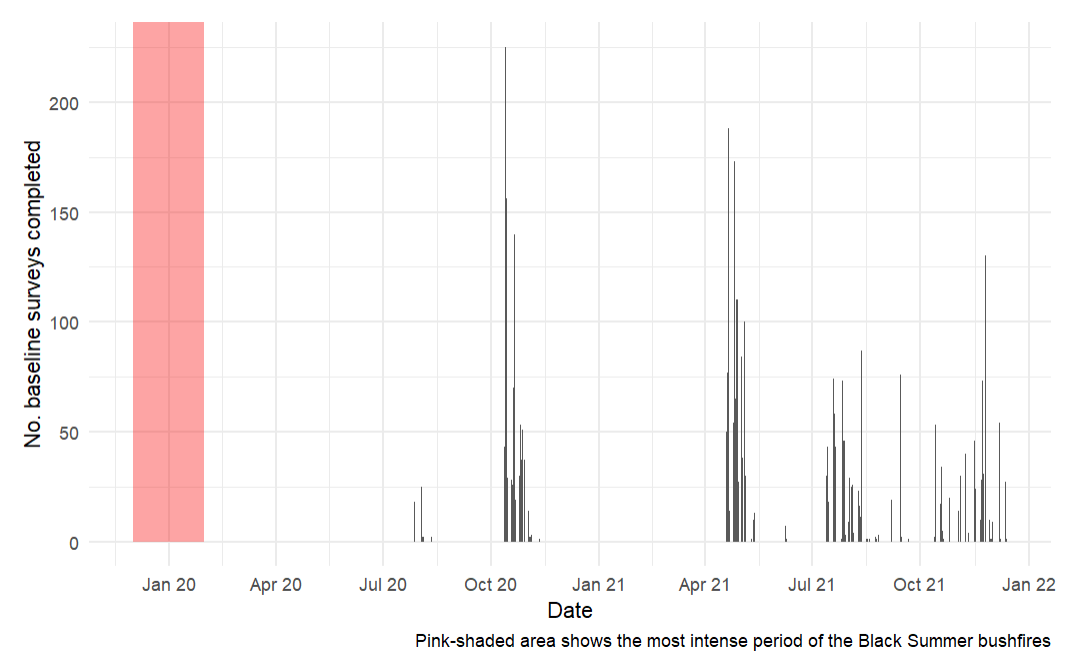


*Supplementary Figure 2: Dates for baseline data collection and Black Summer bushfires*

*Supplementary Table 1: Comparison of initial and final sample characteristics*

|  | **Initial sample (n=6,268)** | **Final sample (n=2,967)** |
| --- | --- | --- |
| **Variable** | **% (n)** | **% (n)** |
| ***Gender*** |  |  |
| Male | 46.1% (2889) | 44.1% (1308) |
| Female | 49.3% (3089) | 52.0% (1542) |
| Another gender | 2.9% (183) | 2.9% (85) |
| Prefer not to say | 1.7% (107) | 1.1% (32) |
| ***Age at baseline*** |  |  |
| 11 years | 0.1% (6) | 0.1% (2) |
| 12 years | 3.7% (231) | 3.9% (115) |
| 13 years | 51.9% (3250) | 53.7% (1592) |
| 14 years | 44.3% (2774) | 42.3% (1256) |
| 15 years | 0.1% (7) | 0.1% (2) |
| ***Sexuality*** |  |  |
| Heterosexual or straight | 69.2% (4340) | 74.4% (2208) |
| Sexuality diverse | 13.2% (828) | 12.7% (376) |
| Not sure | 8.8% (553) | 8.5% (253) |
| Prefer not to say | 4.7% (293) | 4.4% (130) |
| NA | 4.1% (254) | 0.0% (0) |
| ***Symptoms of mental illness*** |  |  |
| Depression symptoms below threshold | 84.8% (5315) | 88.8% (2636) |
| Depression symptoms above threshold | 15.2% (952) | 11.2% (331) |
| NA | 0.0% (1) | 0.0% (0) |
| Anxiety symptoms below threshold | 81.2% (5087) | 84.3% (2502) |
| Anxiety symptoms above threshold | 18.7% (1170) | 15.7% (465) |
| NA | 0.2% (11) | 0.0% (0) |
| Distress symptoms below threshold | 68.3% (4280) | 72.8% (2159) |
| Distress symptoms above threshold | 31.6% (1982) | 27.2% (808) |
| NA | 0.1% (6) | 0.0% (0) |
| Insomnia symptoms below threshold | 88.5% (5548) | 91.5% (2715) |
| Insomnia symptoms above threshold | 11.1% (698) | 8.5% (252) |
| NA | 0.4% (22) | 0.0% (0) |
| Suicidality symptoms below threshold | 87.6% (5488) | 96.0% (2848) |
| Suicidality symptoms above threshold | 4.5% (283) | 4.0% (119) |
| NA | 7.9% (497) | 0.0% (0) |
| Notes: ‘Other’ Gender is an aggregation of ‘Non-binary’ and ‘Other’. 85.3% of participants lived in New South Wales. The thresholds for symptoms of mental illness were taken from validated instruments as described in the text. | | |

*Supplementary Table 2: Specific bushfire harms experienced by bushfire-harmed subsample*

| **Combination of bushfire harms** | **n** | **percent** |
| --- | --- | --- |
| Evacuation only | 101 | 60.5% |
| Property damage only | 5 | 3.0% |
| Injury only | 39 | 23.4% |
| **One type of harm only** | **145** | **86.8%** |
| Evacuation and property damage | 7 | 4.2% |
| Evacuation and injury | 11 | 6.6% |
| Property damage and injury | 0 | 0.0% |
| **Two types of harm** | **18** | **10.8%** |
| Evacuation, property damage and injury | 4 | 2.4% |
| **Three types of harm** | **4** | **2.4%** |
| **Total sample harmed by bushfires** | **167** | **100.0%** |

*Supplementary Table 3: Forced-selection models of 24-month mental health outcomes with standard errors*

|  | **Depression**  **symptoms** | **Anxiety**  **symptoms** | **Distress**  **symptoms** | **Insomnia**  **symptoms** | **Suicidality**  **symptoms** |
| --- | --- | --- | --- | --- | --- |
| **Parameter** | **Coefficient**  **(95%CI)** | **Coefficient**  **(95%CI)** | **Coefficient**  **(95%CI)** | **Coefficient**  **(95%CI)** | **Coefficient**  **(95%CI)** |
| ***Baseline outcome*** |  |  |  |  |  |
| Above threshold at baseline | 1.467  (0.151) | 1.673  (0.131) | 1.453  (0.101) | 1.593  (0.161) | 1.825  (0.289) |
| ***Gender identity*** |  |  |  |  |  |
| Gender identity - female | 0.665  (0.144) | 1.137  (0.153) | 1.404  (0.101) | 0.952  (0.147) | 0.235  (0.276) |
| Gender identity - another gender or prefer not to say | 0.937  (0.273) | 1.314  (0.279) | 1.235  (0.237) | 0.827  (0.315) | 0.676  (0.414) |
| ***Sexuality*** |  |  |  |  |  |
| Sexuality - diverse | 0.427  (0.161) | 0.122  (0.160) | 0.251  (0.125) | -0.224  (0.178) | 1.097  (0.284) |
| Sexuality - not sure | 0.276  (0.212) | 0.074  (0.203) | 0.398  (0.153) | -0.078  (0.223) | 0.940  (0.373) |
| ***Other demographics*** |  |  |  |  |  |
| Do not speak English at home | -0.208  (0.267) | -0.551  (0.282) | -0.406  (0.191) | -0.004  (0.256) | -0.456  (0.557) |
| Perceived SES - low | 0.277  (0.135) | 0.209  (0.130) | 0.132  (0.097) | 0.460  (0.137) | 0.114  (0.257) |
| Perceived SES - prefer not to say | 0.053  (0.208) | 0.106  (0.194) | 0.188  (0.142) | 0.363  (0.202) | 0.052  (0.382) |
| ***Mental health and trauma*** |  |  |  |  |  |
| MH diagnosis - any | 0.463  (0.155) | 0.480  (0.148) | 0.385  (0.127) | 0.388  (0.160) | 0.971  (0.252) |
| Adverse childhood experiences - at least one | 0.462  (0.159) | 0.354  (0.145) | 0.198  (0.101) | 0.450  (0.152) | 0.743  (0.356) |
| COVID - diagnosed or quarantined | 0.144  (0.176) | -0.022  (0.174) | 0.023  (0.134) | 0.032  (0.182) | 0.095  (0.32) |
| Bushfire harm - yes | -0.309  (0.289) | 0.080  (0.249) | 0.223  (0.191) | -0.511  (0.302) | -0.125  (0.499) |
| ***Model predictive power*** |  |  |  |  |  |
| AUC | 0.749 | 0.807 | 0.789 | 0.729 | 0.807 |
| Notes: Predictors have been included in the table if they are significant for any of the five outcomes.  * Significant at p < 0.05. ** Significant at p < 0.01. *** Significant at p < 0.001.  ‘Baseline outcome – Above threshold at baseline’ means that the participant was over the clinical threshold for the condition in that column at the time of their baseline data collection.  In Gender identity, the categories ‘another gender’ and ‘prefer not to say’ were collapsed because of small cell sizes; both had elevated rates of mental illness compared to male and female.  AUC is Area Under Curve. ACEs is Adverse Childhood Events. SES is Socio-Economic Status.  Reference categories: Baseline outcome - below threshold, Gender identity - male, Sexuality - heterosexual, Do speak English at home, Perceived SES - high, MH diagnosis - none, ACEs - none, COVID - neither diagnosed nor quarantined, Bushfire harm - no | | | | | |

*Supplementary Table 4: Comparison of odds ratios for bushfire harm in multivariable fixed-effects models*

|  | **Cross-sectional models**  **Estimate (95% CI)** | **Longitudinal models Estimate (95% CI)** |
| --- | --- | --- |
| Depression | 1.42 (1.12-1.71)* | 0.734 (0.402-1.258)^n.s.^ |
| Anxiety | 0.39 (0.12-0.66)* | 1.084 (0.653-1.738)^n.s.^ |
| Distress | n.s. | 1.250 (0.856-1.811)^n.s.^ |
| Insomnia | 1.63 (1.33-1.92)** | 0.600 (0.318-1.048)^n.s.^ |
| Suicidality | 1.98 1.57-2.39)** | 0.883 (0.292-2.142)^n.s.^ |

Notes: ** significant at *p* < .007. * significant at *p* ≤ .05. n.s. Not significant. Cross-sectional models estimates from Beames et al (Model 5 in each of Tables S6 to S10)

(Beames et al., 2023).
